# StenM_174: A Novel Podophage That Infects a Wide Range of *Stenotrophomonas* spp. and Suggests a New Subfamily in the Family *Autographiviridae*

**DOI:** 10.3390/v16010018

**Published:** 2023-12-21

**Authors:** Vera V. Morozova, Vyacheslav I. Yakubovskij, Ivan K. Baykov, Yuliya N. Kozlova, Artem Yu. Tikunov, Igor V. Babkin, Alevtina V. Bardasheva, Elena V. Zhirakovskaya, Nina V. Tikunova

**Affiliations:** 1 Laboratory of Molecular Microbiology, Institute of Chemical Biology and Fundamental Medicine Siberian Branch of Russian Academy of Sciences, Novosibirsk 630090, Russia; yakubovskij97@gmail.com (V.I.Y.); arttik1986@gmail.com (A.Y.T.); i_babkin@mail.ru (I.V.B.);; 2 Faculty of Natural Sciences, Novosibirsk State University, Novosibirsk 630090, Russia; 3 Shared Research Facility “Siberian Circular Photon Source” (SRF “SKIF”) of Boreskov Institute of Catalysis SB RAS, Novosibirsk 630090, Russia

**Keywords:** *Autographiviridae*, *Stenotrophomonas maltophilia*, virulent phage, comparative genomics, tail spike, receptor-binding protein, structural modeling, AlphaFold

## Abstract

*Stenotrophomonas maltophilia* was discovered as a soil bacterium associated with the rhizosphere. Later, *S. maltophilia* was found to be a multidrug-resistant hospital-associated pathogen. Lytic bacteriophages are prospective antimicrobials; therefore, there is a need for the isolation and characterization of new *Stenotrophomonas* phages. The phage StenM_174 was isolated from litter at a poultry farm using a clinical strain of *S. maltophilia* as the host. StenM_174 reproduced in a wide range of clinical and environmental strains of *Stenotrophomonas*, mainly *S. maltophilia*, and it had a podovirus morphotype. The length of the genomic sequence of StenM_174 was 42,956 bp, and it contained 52 putative genes. All genes were unidirectional, and 31 of them encoded proteins with predicted functions, while the remaining 21 were identified as hypothetical ones. Two tail spike proteins of StenM_174 were predicted using AlphaFold2 structural modeling. A comparative analysis of the genome shows that the *Stenotrophomonas* phage StenM_174, along with the phages Ponderosa, Pepon, Ptah, and TS-10, can be members of the new putative genus *Ponderosavirus* in the *Autographiviridae* family. In addition, the analyzed data suggest a new subfamily within this family.

## 1. Introduction

*Stenotrophomonas maltophilia* is a ubiquitous Gram-negative bacterium that is often isolated from the rhizosphere and plant surfaces. It belongs to the *Stenotrophomonas* genus of the *Xanthomonadaceae* family. The ability to utilize a wide range of carbon sources and resistance to heavy metals enable *S. maltophilia* to survive under various adverse conditions [1]. *S. maltophilia* strains are not phytopathogens; moreover, they are able to produce plant growth hormone (indole-3-acetic acid) and metabolize phenols and xenobiotics [2]. These properties make *S. maltophilia* a prospective candidate for application in agriculture and soil bioremediation. However, the ability of *S. maltophilia* to cause disease in humans prevents this bacterium from being used for human benefit.

The above-mentioned properties allow *S. maltophilia* to spread in a hospital environment. In addition, surface structures such as LPS, flagella, pili, and fimbriae help *S. maltophilia* to attach to the surfaces of medical equipment and form biofilms; therefore, this bacterial species is listed as a nosocomial pathogen worldwide [3]. The main factors contributing to *S. maltophilia* infection are weakened immunity, chronic respiratory diseases, cancer, and prolonged stay in the hospital or intensive care unit [4]. Patients with cystic fibrosis are at greater risk of *S. maltophilia* infection than the general population [5]. This pathogen is most commonly associated with respiratory infections and is the causative agent of severe bacteremia, meningitis, endocarditis, pneumonia, osteomyelitis, endophthalmitis, and catheter-related septicemia [6].

The main problem in the treatment of infections caused by *S. maltophilia* is their natural resistance to various classes of antibiotics, namely β-lactams, aminoglycosides, tetracyclines, macrolides, chloramphenicol, and polymyxins. The insusceptibility of *S. maltophilia* is due to many intrinsic and acquired mechanisms, including reduced membrane permeability, more than a dozen described efflux pumps, β-lactamases, and aminoglycoside-modifying enzymes. In addition, tolerance to antiseptics and hydrogen peroxide-based disinfectants is provided by the presence of the *qacEΔ1* and *katA* genes in the majority of isolates. This gives *S. maltophilia* advantages for persistence and spread in hospital settings [6,7,8]. The main recommended antibiotics for infections caused by *S. maltophilia* are trimethoprim/sulfamethoxazole and cefiderocol (https://www.eucast.org/ast_of_bacteria/calibration_and_validation, access on 18 October 2023), and although sensitivity remains high, resistance to these antibiotics is increasing [9]. Pathogenicity and prevalence, combined with high levels of antimicrobial resistance, necessitate alternative methods of treating infections caused by *S. maltophilia*, and phage therapy is one of the promising treatments.

The first *S. maltophilia* bacteriophage, M6, was isolated in 1973 [6]. At that time, phages were considered genetic tools used to map the genome of their bacterial hosts using transduction. Currently, most phages are isolated and characterized from the point of view of therapeutic use. To date, 65 complete genomes of *S. maltophilia* phages are presented in the NCBI GenBank database (https://www.ncbi.nlm.nih.gov/nucleotide, accessed on 3 October 2023). Five of them are members of the family *Inoviridae*, which includes filamentous bacteriophages with circular single-stranded DNA (Data S1). The remaining 60 phage genomes belong to the class Caudoviricetes, which contains tailed phages with linear double-stranded DNA. They are diverse in the genome content, life cycle, and morphotype [10,11,12,13,14,15,16]. According to the last ICTV release [17], the majority of *Stenotrophomonas* phages were classified as members of the *Autographiviridae* (11 phages), *Mesyanzhinovviridae* (6 phages), *Schitoviridae* (5 phages), *Straboviridae* (2 phages), *Casjensviridae* (1 phage), and *Peduoviridae* (1 phage) families. In addition, *Stenotrophomonas* phages were assigned to the subfamily *Beaumontvirinae* (six phages), and to several genera, namely *Menderavirus* (seven phages), *Delepquintavirus* (three phages), and *Septimatrevirus* (two phages) (https://www.ncbi.nlm.nih.gov/taxonomy, accessed on 3 October 2023) (Data S1). A total of 16 *Stenotrophomonas* bacteriophages remain unclassified and can be considered members of new genera/subfamilies/families within the class Caudoviricetes. About half of these phages (*n* = 22) have the podovirus morphotype; they include members of the families *Autographivirinae, Schitoviridae*, and four unclassified members of the class Caudoviricetes. According to the GenBank database (https://www.ncbi.nlm.nih.gov/nucleotide, accessed on 3 October 2023), almost all the previously studied phages have *S. maltophilia* as a host, and only four phages are specific to unidentified *Stenotrophomonas* sp. (Data S1). 

The diversity of *S. maltophilia* phages is useful for the development of effective phage cocktails and the study of novel mechanisms of phage biology. The first successful application of the IME-SM1 phage against *S. maltophilia* infection in a mouse model was reported in 2013 [18]. Later, the activity of the phages BUCT603 and BUCT609 against *S. maltophilia* infections was studied in mice [19,20]. In addition, one more phage, BUCT700, has been used to fight *S. maltophilia* infection in the *Galleria mellonella* model [21]. Recently, CUB19 was applied to eradicate biofilm, formed by *S. maltophilia* [22]. An example of an unusual application of *Stenotrophomonas* phages is their use to prevent the growth of the corrosion-producing *S. maltophilia* [23]. However, the heterogeneity and resistance of *S. maltophilia* isolates necessitate the search for new *Stenotrophomonas* phages with a wide host range and high lytic activity. 

In this study, a novel *Stenotrophomonas* phage, StenM_174, was isolated, which infected a wide range of clinical and environmental strains of *Stenotrophomonas*, mainly *S. maltophilia*. StenM_174 had a podovirus morphotype and possessed high lytic properties. The genome of StenM_174 contained linear dsDNA with a length of 42,956 bp, and it encoded 52 putative unidirectional genes. The structure of the receptor-binding proteins of StenM_174 was predicted using AlphaFold2. A comparative analysis of the genome showed that the *Stenotrophomonas* phage StenM_174, along with the phages Ponderosa [24], Pepon [25], Ptah [26], and TS-10 (GenBank ID OK018136), can be members of the new putative genus *Ponderosavirus* within the *Autographiviridae* family. In addition, the analyzed data suggest a new subfamily within this family.

## 2. Materials and Methods

### 2.1. Bacterial Strain Identification

The clinical isolate of *S. maltophilia* was kindly provided by the Novosibirsk Research Institute of Traumatology and Orthopedics. The identification of bacterial species was performed by sequencing a fragment of the 16S rRNA gene with a length of 1308 bp. Primers 16s-8-f-B 5′-AGRGTTTGATCCTGGCTCA-3′ and 16s-1350-r-B 5′-GACGGGGCGGTGTGTACAAG-3′ were used for amplification and sequencing as described previously [27]. The PCR protocol contained 33 cycles of amplification, and each cycle included denaturation (95 °C, 5 min), annealing (55 °C, 30 s), and elongation (72 °C, 1 min). Sequencing reactions were performed using a BigDye Terminator v.3.1 Cycle Sequencing Kit (Applied Biosystems, Foster City, CA, USA) according to the manufacturer’s instructions. Nucleotide sequences were obtained using an ABI 3500 Genetic Analyzer (Applied Biosystems, Foster City, CA, USA) and compared with sequences of the 16S rRNA genes from the NCBI GenBank database. The bacterial strain was deposited as *S. maltophilia* CEMTC 2355 in the Collection of Extremophilic Microorganisms and Type Cultures (CEMTC) of the Institute of Chemical Biology and Fundamental Medicine SB RAS, Novosibirsk, Russia.

### 2.2. Phage Isolation and Propagation

To obtain the phages, 5 g of litter from a poultry farm was mixed with 10 mL of sterile phosphate-buffered saline (PBS), pH 7.5, and centrifuged at 8000× *g* for 10 min. Then, the obtained supernatant was sterilized through a 0.22 μm filter (Wuxi Nest Biotechnology, Wuxi, China). Screening for the presence of phages was carried out by dropping 10 μL aliquots of filtrate onto a freshly prepared lawn of *S. maltophilia* CEMTC 2355 in the top agar (Becton, Dickinson, and Company Sparks Difco Laboratories, Franklin Lakes, New Jersey, USA). Plates were incubated overnight at 37 °C. The revealed phage plaques were cut from the top agar, suspended in sterile PBS, and incubated with shaking overnight to extract the phages from the agar. The next day, tenfold dilutions of the phage-containing eluate were dropped onto a fresh layer of *S. maltophilia* CEMTC 2355 to obtain single plaques. These plaques were used for subsequent phage extraction; three cycles of phage dilution–extraction were performed.

To amplify StenM_174, 50 mL of exponentially growing *S. maltophilia* CEMTC 2355 in the Lysogeny Broth, LB (BD Difco, Franklin Lakes, NJ, USA) were infected with the phage; the multiplicity of infection (MOI) was 0.1. The infected culture was incubated with shaking at 37 °C until the bacterial lysis occurred. Afterward, the bacterial lysate was centrifuged at 10,000× *g* for 30 min, and phage particles were purified from the supernatant using polyethylene glycol 6000 (AppliChem, Darmstadt, Germany) precipitation as described previously [28]. The phage-containing precipitate was dissolved in 500 µL of STM buffer (0.59 g of NaCl; 7.88 g of Tris-HCl, pH 7.5; and 2.38 g of MgCl_2_ per 1 L).

### 2.3. Phage Plaques and Phage Particle Morphology

The morphology of the StenM_174 plaques was detected on the lawn of a sensitive culture of *S. maltophilia* CEMTC 2355. Plaques were examined after the incubation of plates overnight at 37 °C. The morphology of the phage particles was determined using transmission electron microscopy with preliminary negative staining as described previously [29].

### 2.4. Biological Properties and Host Range Assay

The biological properties of StenM_174 were studied using the *S. maltophilia* CEMTC 2355 as the host. All experiments were performed twice, each in three technical repeats. Graphs and statistical analysis were performed in GraphPad Prizm v. 8.0.1.

To determine the burst size for StenM_174, 10 mL of exponentially growing bacteria were centrifuged at 8000× *g* for 10 min. Then, the pellet was resuspended in 500 μL of LB and the phage StenM_174 was added to the cells with an MOI of 0.001. The culture was incubated for 5 min without shaking at 37 °C; after that, cells were pelleted via centrifugation, the supernatant with non-adsorbed phages was removed, and the bacterial pellet was dissolved in 10 mL of LB. The infected bacterial culture was incubated with shaking at 37 °C for 30 min, and aliquots were taken every 2.5 min and used for phage titer determination. The latent period and burst size of StenM_174 were calculated based on the obtained data.

Phage adsorption experiments were performed as described previously [29]. Briefly, the exponentially growing *S. maltophilia* CEMTC 2355 was infected with StenM_174 to a final concentration of 10^5^ plaque-forming units per mL (pfu/mL). The infected culture was incubated with shaking at 37 °C for 30 min, and aliquots were taken every minute to determine the titer of free phages.

The lytic activity of StenM_174 was analyzed as described previously [30], with minor modifications. Briefly, the phage was added to exponentially growing bacterial cultures of *S. maltophilia* CEMTC 2355 in three different MOIs, 0.1, 0.01, and 0.001. Then, the phage-containing cultures were incubated without shaking at 37 °C for 30 min to improve phage infection. Next, the infected cultures were incubated at 37 °C with shaking, and aliquots were taken every 30 min to determine the bacterial titer in the phage lytic life cycle. Based on the obtained data, the multistep killing curves of bacteria in the life cycle of StenM_174 were calculated.

The host range for StenM_174 was determined using the spot-assay method, as described previously [31]. A total of 65 strains of *Stenotrophomonas* spp. from the CEMTC ICBFM SB RAS were tested, which included clinical and environmental strains, as well as strains isolated from insects. To determine the titer of the phage on a sensitive bacterial strain, tenfold dilutions of a phage-containing suspension were dripped onto a fresh layer of appropriate bacteria in the top agar to obtain single plaques, and the phage titer was calculated the next day. The bacterial susceptibility to StenM_174 was evaluated based on the efficacy of plating (EOP) as previously described [22]. 

### 2.5. Genome DNA Purification and Complete Genome Sequencing

Genome DNA purification was carried out as described previously [32]. Briefly, DNase and RNase (Thermo Fisher Scientific, Waltham, MA, USA) were added to phage suspension, each to a final concentration of 5 μg/mL, and the mixture was incubated at 37 °C for 30 min. Next, phage suspension was supplemented with 1/25 volume of 0.5 M EDTA (pH 8.0), 1/20 volume of 10% solution of SDS, and proteinase K (Thermo Fisher Scientific, Waltham, MA, USA) to a final concentration of 100–200 μg/mL. Then, the suspension was incubated at 55 °C for 3 h, and phage DNA was purified via phenol–chloroform extraction. Furthermore, ethanol supplemented with 1/30 volume of 3 M sodium acetate (pH 4.8) was added to precipitate DNA. The phage DNA quality was estimated through agarose gel electrophoresis. Covaris Ultrasonicator (Covaris, Woburn, MA, USA) was applied for phage DNA fragmentation, and the obtained DNA fragments were purified on magnetic beads. An NEB Next Ultra II DNA Library Prep Kit for Illumina and NEB Next Multiplex Oligos for Illumina (both from New England BioLab, Ipswich, MA, USA) were used for DNA library construction. Sequencing was performed using the MiSeq Benchtop Sequencer and MiSeq v. 2 Reagent Kit (2 × 250 base reads) (Illumina Inc., San Diego, CA, USA). Trimmomatic tool v. 0.39 [33] was used to check the obtained data for quality and remove adapter sequences. The phage genome was assembled de novo using SPAdes Genome Assembler v. 3.15.4 (http://cab.spbu.ru/software/spades, accessed on 2 July 2023) [34]. The sequencing coverage was estimated to be 1395.

### 2.6. Analysis of Phage Genome 

Rapid Automated Annotation Service (RAST) v. 2.0 [35] (https://rast.nmpdr.org, accessed on 20 July 2023) was used for the StenM_174 genome analysis. Then, the annotation was validated manually with BLASTX search against sequences deposited in the NCBI GenBank database (https://ncbi.nlm.nih.gov, accessed on 25 July 2023). In addition, InterProScan and HHpred tools were used for protein function identification [36,37]. Phage genome termini and DNA packaging strategy were determined using the PhageTerm tool v. 1.0.12 [38]. Signal sequences in proteins were detected using SignalP 6.0 [39]. The genome of StenM_174 was deposited to the NCBI GenBank database with the accession number OR729839.

To estimate the taxonomy of StenM_174, a comparative proteomic phylogenetic analysis was performed on the Viral Proteome Tree Server v. 3.7 (ViPTree) [40] (https://www.genome.jp/viptree, accessed on 23 September 2023). Intergenomic similarity (SG) was calculated using the Virus Intergenomic Distance Calculator (VIRIDIC) [41] (http://rhea.icbm.uni-oldenburg.de/VIRIDIC, accessed on 25 September 2023). 

### 2.7. Phylogenetic Analysis of Phage Proteins

Proteins of interest were searched in the NCBI GenBank database with BLASTP and BLASTX (https://blast.ncbi.nlm.nih.gov/, accessed on 3 October 2023) and extracted for further analysis. Protein sequences were aligned, and phylogenetic analysis was performed in the MEGA 7.0 software [42].

### 2.8. Modeling of 3D Structures of Tail Spike Proteins

Protein structures were modeled using the ColabFold v. 1.5.3 implementation of AlphaFold2 and AlphaFold-Multimer [43] (https://colab.research.google.com/github/sokrypton/ColabFold/blob/main/AlphaFold2.ipynb, accessed on 8 November 2023). The models were edited and visualized using UCSF Chimera v. 1.13 [44]. Molecular dynamics (MD) was used for the relaxation of individual trimeric domains of the gp43 protein. MD experiments were performed using GROMACS 2020.3 [45] running on Nvidia Tesla V100-equipped GPU nodes of the High-Performance Computing Center of Novosibirsk State University (“NUSC NSU”). MD experiments were performed for 50 ns at 310 K and 1 bar pressure using the amber99SB force field and tip3p water molecules. Simulation trajectories were centered using GROMACS and analyzed using VMD v. 1.9.3.

## 3. Results

### 3.1. Bacterial Host Identification

The clinical isolate *S. maltophilia* CEMTC 2355 was kindly provided by the Novosibirsk Research Institute of Traumatology and Orthopedics. The strain was mesophilic and grew at 25 °C and above, and an optimum growth temperature was 37 °C. Light-field microscopy revealed motile small bacilli. The identification of bacterial species was carried out via the sequencing of the 16S rRNA gene; the sequence was submitted to the NCBI GenBank database (ID OP393915).

### 3.2. StenM_174 Isolation, Plaques, and Phage Particle Morphology

**Figure 1:**
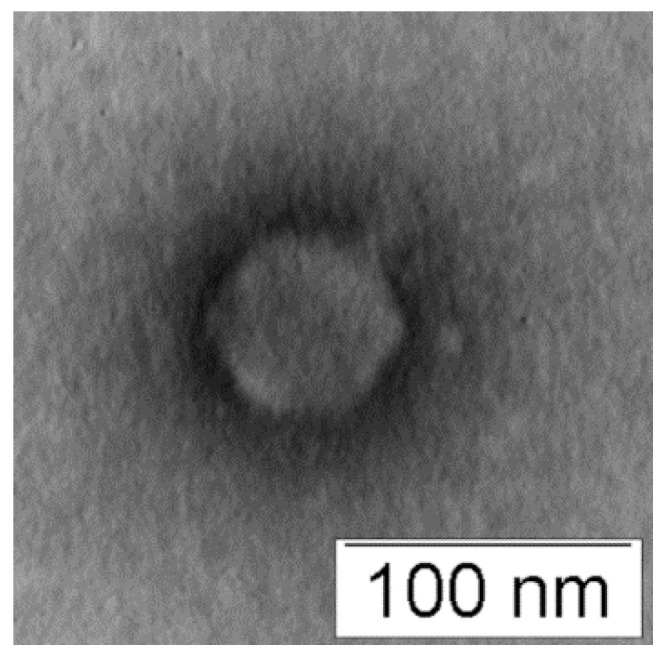
Electron micrograph of StenM_174 phage particles negatively stained with 1% uranyl acetate.

StenM_174 was isolated from a litter sample obtained from a poultry farm using a clinical strain of *S. maltophilia* CEMTC 2355 as the host. StenM_174 formed large transparent plaques with a diameter of about 1 mm on the lawn of the host strain. The electron microscopy of StenM_174 revealed an icosahedral head (Ø ~60 nm) connected to a short non-contractile tail (Figure 1). The morphology of the phage particle corresponded to the morphotype of podoviruses.

### 3.3. StenM_174 Biological Properties and Host Range

**Figure 2:**
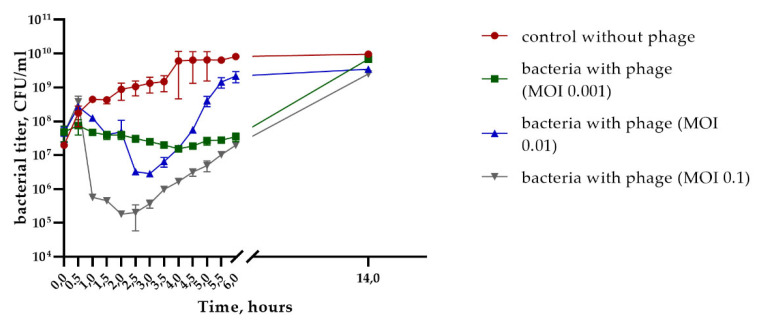
The multistep bacterial killing curve for the *S. maltophilia* CEMTC 2355 in the life cycle of StenM_174.

The biological properties of StenM_174 were studied using the clinical strain *S. maltophilia* CEMTC 2355 as the host. More than 64% of StenM_174 phage particles attached to host cells within 9 min in phage adsorption experiments. A one-step growth assay of StenM_174 revealed a short latent period of about 20 min with a burst size of ~ 100 phage particles per infected cell. The multistep bacterial killing experiments were carried out in three variants, in which phage was added to cells with MOIs of 0.1, 0.01, and 0.001; bacterial lysis was dose-dependent, and the MOI value of 0.1 corresponded to the fastest and maximum decrease in the number of viable host cells (Figure 2). The obtained data showed the virulent properties of StenM_174.

The host range of StenM_174 was analyzed using 65 strains of 10 *Stenotrophomonas* species (Table 1). StenM_174 was specific mainly to *S. maltophilia* strains, as it infected 16 of the 21 tested strains. No correlation was found between the origin of this particular *S. maltophilia* strain and its sensitivity to the phage (Table 2). In addition, several environmental strains of *S. rhizophila* and *S. lactitubi* were sensitive to this phage (Table 1 and Table 2). It is noteworthy that StenM_174 reproduces well in the temperature range from 25 °C to 37 °C (Table 2). Its reproduction likely depends on the metabolic activity of a particular bacterial host at a certain temperature.

### 3.4. Genome Characteristics

**Figure 3:**
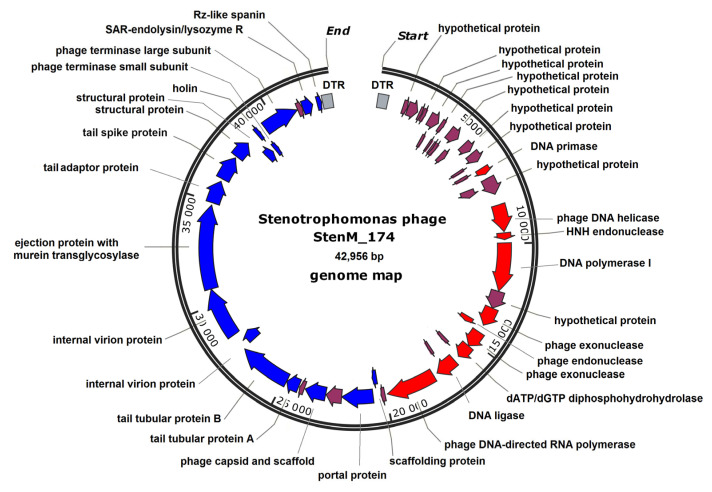
StenM_174 genome map. Hypothetical genes are marked with dark red arrows, DNA–RNA metabolism genes are marked with red arrows, and late genes are marked with blue arrows; direct terminal repeats are marked with gray boxes.

The length of the genome of the phage was 42,956 bp with a GC content of 59.9%, which is close to ~66.5% GC content in *S. maltophilia* genomes (GenBank IDs NC011071 and AM743169). The termini of the genome were redundant and contained direct terminal repeats with a length of 499 bp (Data S2). A total of 52 putative open reading frames (ORFs) were identified using the RAST tool; of those, 31 encoded proteins with predicted functions, while the remaining 21 ORFs were defined as hypothetical ones. No genes encoding tRNA were found. All ORFs were unidirectional and could be grouped into clusters according to their putative functions; however, the precise definition of the boundaries between genetic clusters is complicated due to the presence of several genes that encode hypothetical proteins (Figure 3).

A cluster of 15 hypothetical genes is located at the beginning of the genome. Only two of their gene products, gp12 and gp13, have limited similarity with the shock protein A and S-adenosyl methionine lyase of the *Escherichia coli* phage K12, respectively. Presumably, this cluster of genes, comprising the so-called early genes, is responsible for switching the bacterial host cell to the synthesis of phage RNA and phage proteins. The next cluster of genes is associated with the synthesis of phage DNA and RNA. It includes the genes encoding phage DNA primase (ORF 16), phage-associated DNA helicase (ORF 20), DNA polymerase (ORF 22), DNA ligase (ORF 29), and DNA-dependent RNA polymerase (ORF 31). One more group of genes is responsible for virion assembly, DNA packaging, and the phage-induced lysis of the infected cell. The putative structural protein gp42 was annotated as an ejection protein with murein transglycosylase activity. In addition, this cluster contained genes encoding the proteins of lytic cassette, and one of them, gp51, was classified as a peptidoglycan N-acetylmuramoyl hydrolase. The signal peptide was detected in the gp51 sequence using the SignalP 6.0 tool; hence, gp51 is a SAR-endolysin (Data S3).

The characteristics of the genome of StenM_174, including the size of the genome, the presence of direct terminal repeats, and single-subunit RNA and DNA polymerases, attributed this phage to the *Autographiviridae* family.

### 3.5. Structural Model of Receptor-Binding Proteins of StenM_174

The analysis of the StenM-174 genome revealed four genes encoding putative tail proteins (gp43–gp46). HHPred and InterProScan search did not reveal homology or function similarity for these proteins, with the exception of gp44, in which a carbohydrate-binding domain was identified. Similar *Stenotrophomonas* podophage Ponderosa has been previously described [24]. Its genome contained four genes, whose products were similar to gp43–gp46 of StenM_174. These four Ponderosa proteins were annotated as tail fiber proteins [24]. In addition, several *Xanthomonas* podophages (NC_020205, NC_048703, and MK903278) contained similar genes, encoding proteins that were annotated as tail fiber proteins. To clarify the putative function of structural proteins gp43–gp46, structural modeling using ColabFold v1.5.3 (a version of AlphaFold2) was performed [43]. 

Three-dimensional (3D) structures of four proteins, gp43–gp46, were modeled. The resulting structures of gp45 and gp46 proteins looked like globular domains and demonstrated no structural similarity with known tail fiber or tail spike proteins (Figure S1). Therefore, further analysis was carried out for proteins gp43 and gp44.

**Figure 4:**
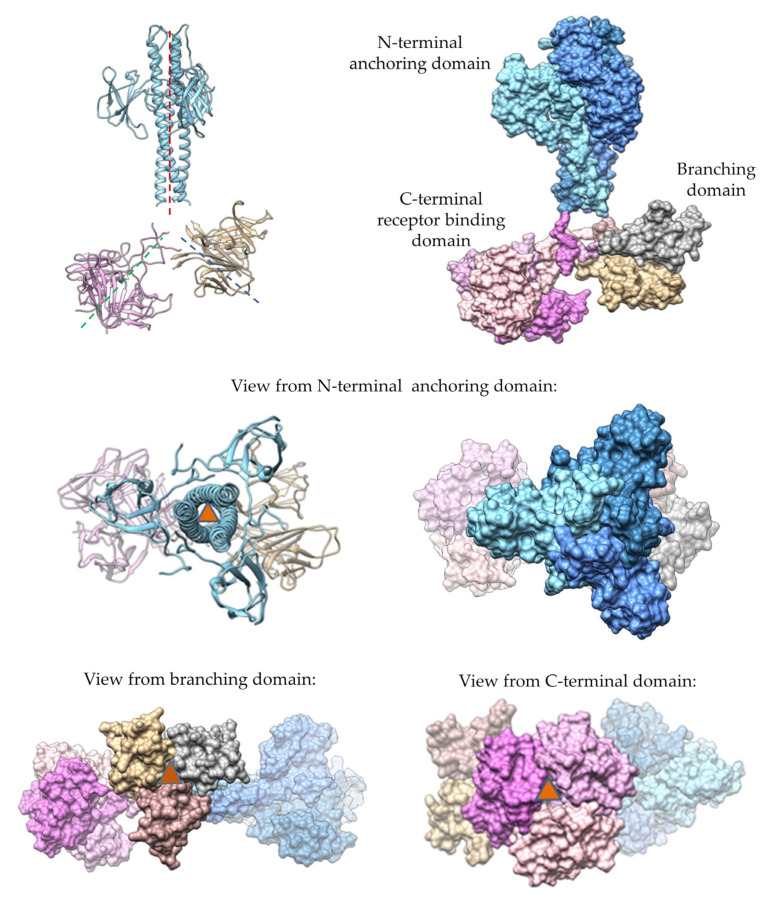
Structural model of homotrimeric form of gp43. N-terminal domains are shown in blue; branching domains are in tan, gray, or brown; and C-terminal domains are in pink. Dashed lines and red triangles indicate 3-fold symmetry axes.

The 3D structure of the homotrimeric form of gp43 was constructed. Globular domains were modeled with high confidence. The extended flexible regions, which connect the globular domains, were modeled with less confidence than globular domains; however, the structure of the gp43 trimer seems reliable (Figure 4). The molecular dynamic relaxation of the structure for 50 ns did not reveal any significant deviations from the initial model during the simulation. The structure of the gp43 trimer was asymmetric, unlike most tail spike/tail fiber proteins, which usually form symmetrical homotrimers. However, an asymmetric type of structure is common for adaptor tail spike proteins that contain T4gp10-like branching domains (brDs) [46,47]. Despite the absence of a general symmetry for the entire molecule, each of the three domains has its own local three-fold axis of symmetry (Figure 4). The N-terminal domain is an anchor (or adapter) domain similar to the N-terminal domain of the gp17 tail fiber protein of the T7 phage (pdb 7EY9, 8E4G, and 8DSP). The N-terminal anchor domain is followed by brD, which is used to attach other tail spikes or tail fibers. The C-terminal domain has a globular structure, which is presumably used for carbohydrate binding and cleavage (Figure 4). The analysis using DALI [48] (http://ekhidna2.biocenter.helsinki.fi/dali, access date 7 November 2023) revealed some structural similarity with one of the domains of the gp9 protein of the T4 phage (pdb 1ZKU, Z-score 8.2). Thus, structural modeling confirmed that the gp43 protein of StenM_174 is a tail spike adaptor protein.

**Figure 5:**
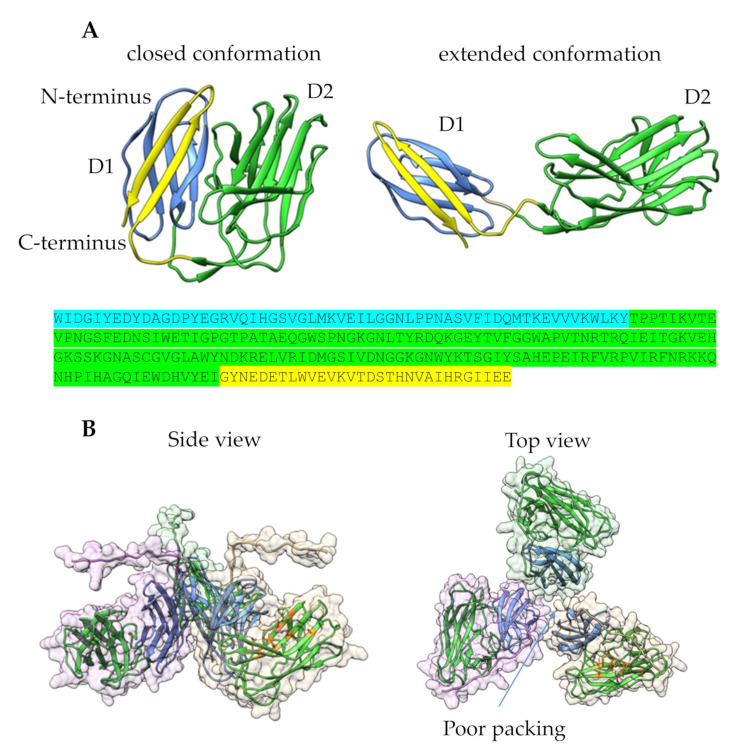
Structural models of gp44 of StenM174: (**A**) model of an N-terminal part of gp44 monomer; the D1 domain is shown in blue and yellow, and the D2 domain is green; the amino acid sequence of D1 and D2 domains are colored corresponding to the 3D structure; (**B**) homotrimeric model of gp44; carbohydrate-binding residues for one D2 domain are shown in orange.

Then, the 3D structures of the gp44 monomer and homotrimer were investigated. The N-terminal part, excluding the first 21 aa, was modeled with high confidence and divided into two domains: D1 with beta-sandwich fold and D2 with jelly roll fold (Figure 5A). Both open and closed conformations were modeled with high confidence. According to the HHpred and DALI analysis, the D2 domain has a structure similar to the carbohydrate-binding domains. The carbohydrate-binding aa residues are located on the concave side of this domain (Figure 5B). The fact that some phage tail spike proteins also contain domains similar to D2 (e.g., gp52 of *Klebsiella* phage Kp7, pdb 7XYC) clearly indicates that gp44 is actually a tail spike protein. The C-terminal part of this protein and N-terminal 21 aa were modeled with low confidence due to the limited number of similar sequences in databases.

According to the results of structural modeling, only gp43 and gp44 were identified as tail spike proteins, and the remaining two, gp45 and gp46, were undefined structural proteins. Thus, StenM_174 has only two putative tail spike proteins: gp43 and gp44.

### 3.6. Comparative Analysis of the StenM_174 Genome and its Taxonomy

**Figure 6:**
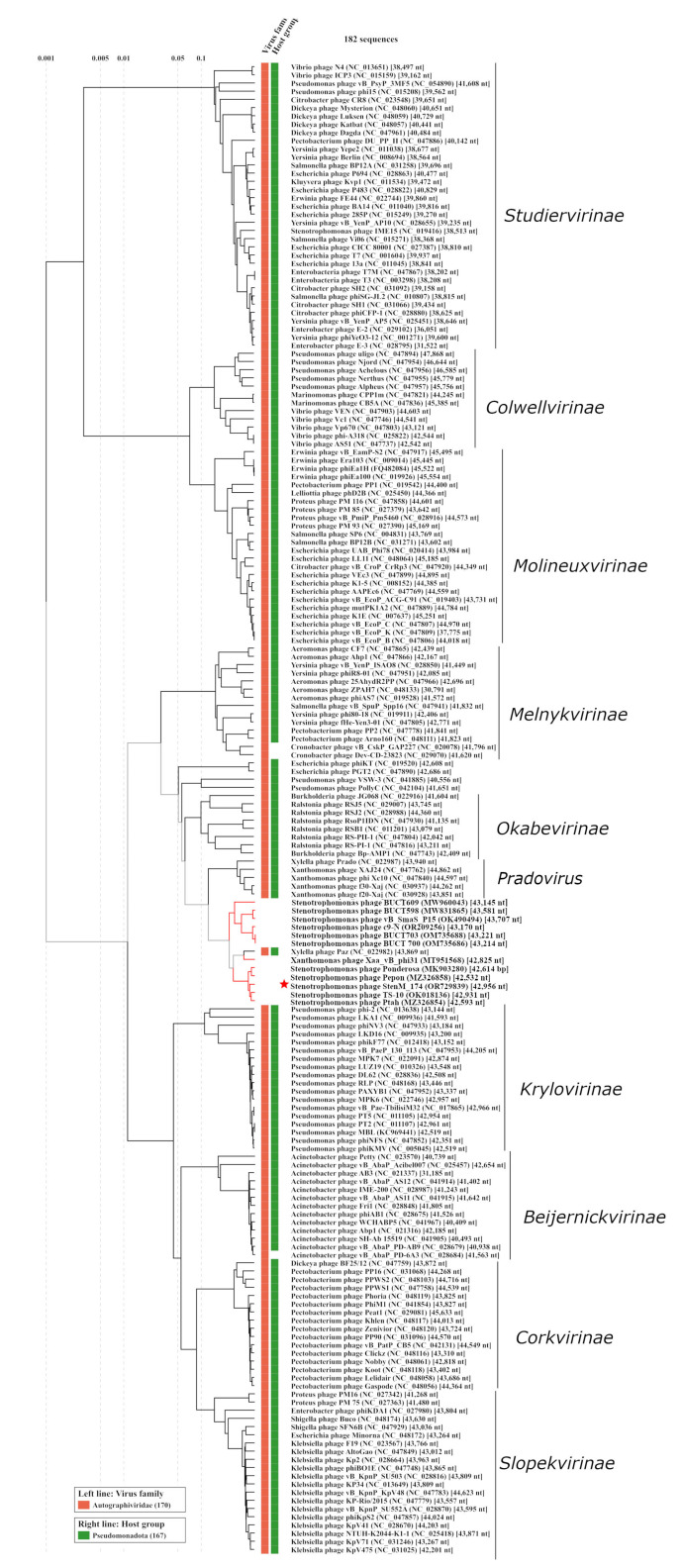
Viral proteomic tree for the phage StenM_174 constructed using ViPTree tool. Red branches indicate phage sequences that were downloaded from the NCBI GenBank database and added to the analysis manually. StenM_174 is marked with a red asterisk.

To clarify the taxonomy of StenM_174, its genome was compared with the available phage sequences from the NCBI GenBank database using BLASTN search (accessed on 20 September 2023). Eleven similar phage genomes were extracted from the NCBI GenBank and used for further comparative analysis with ViPTree. As a result, StenM_174 was assigned to a heterogeneous group of phages, which contained several *Stenotrophomonas* phages, *Xylella* phage Paz, and *Xanthomonas* phage Xaa_vB_phi31 (Figure 6).

**Figure 7:**
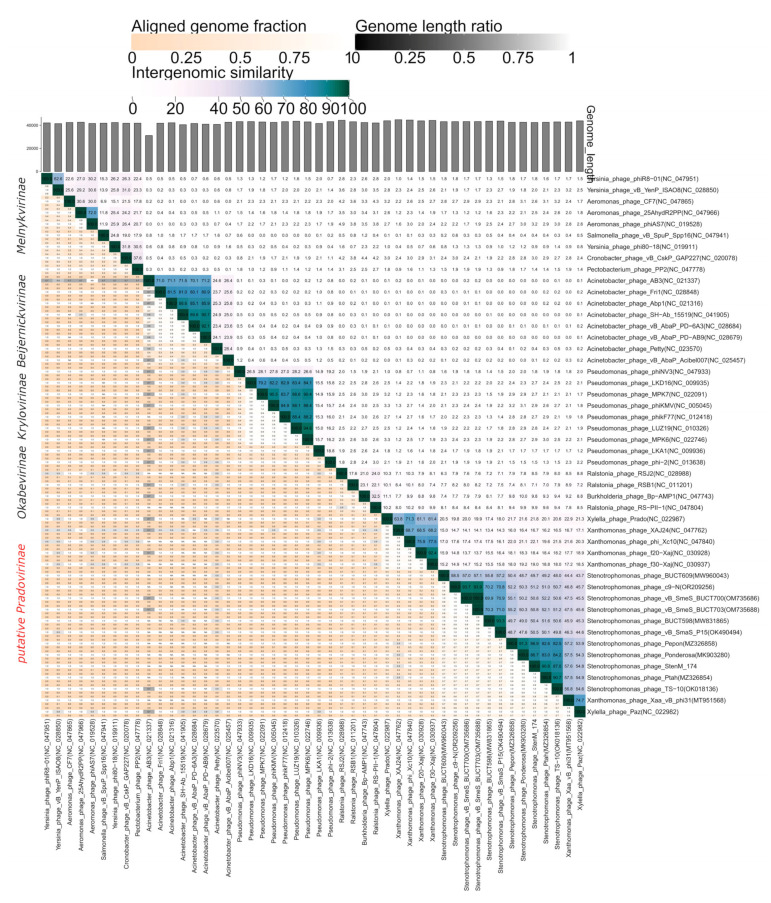
Matrix of intergenomic similarity, calculated for StenM_174 and similar phages.

**Figure 8:**
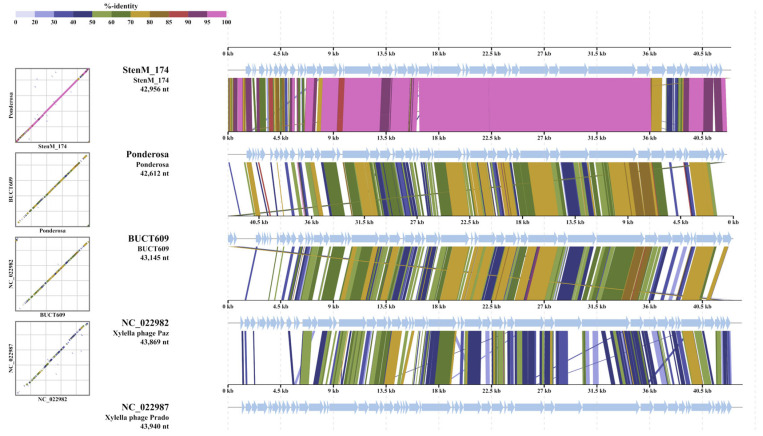
Alignment of the StenM_174 genome together with similar genomes of other phages.

In addition, the analysis of intergenomic similarity for StenM_174 with the most similar phages was carried out using VIRIDIC, and genome alignment was performed using the ViPTree tool. It was found that the level of SG between studied genomes corresponded to the data obtained using ViPTree analysis (Figure 7), and the genomes of the studied phages demonstrated a clear gene synteny (Figure 8). 

Thus, the analyzed group of phages (*Stenotrophomonas* phages, *Xylella* phage Paz, and *Xanthomonas* phage Xaa_vB_phi31) can be divided into three putative genera. The first of them combines *Stenotrophomonas* phages Ponderosa, Pepon, StenM_174, TS-10, and Ptah. The second contains *Stenotrophomonas* phages vB_SmaS_P15, BUCT598, c9-N, vB_SmeS_BUCT703, and vB_SmeS_BUCT700. The *Xylella* phage Paz and the *Xanthomonas* phage Xaa_vB_phi31 can be united into one more genus. All these putative genera have >70% intergenomic similarity between their members (Figure 7), which meets the criteria of definition phage genera established by the ICTV taxonomy committee [49]. It should be noted that the taxonomic position of another *Stenotrophomonas* phage, BUCT609, remains unclear since its level of intergenomic similarity with related phages is <60%; thus, it is likely a prototype of another putative genus. 

We propose to name one of these new genera, including the studied phage StenM_174 and the phages Ponderosa, Pepon, Ptah, and TS-10, *Ponderosavirus*, in accordance with the name of the first studied member of this genus, the Ponderosa phage [24].

Moreover, it was assumed that these three putative genera, together with members of the *Pradovirus* genus, can be combined into a putative subfamily in the *Autographiviridae* family. Currently, the *Autographiviridae* family is a large taxonomy subdivision, which contains more than 380 phages. This family is divided into nine subfamilies, namely *Beijerinckvirinae, Colwellvirinae, Corkvirinae, Krylovirinae, Melnykvirinae, Molineuxvirinae, Okabevirinae, Slopekvirinae,* and *Studiervirinae* [17]. To test our assumption, members of all these subfamilies, along with the studied group of phages, were compared using the ViPTree tool. It was revealed that the investigated *Stenotrophomonas, Xanthomonas*, and *Xylella* phages form a monophyletic clade that is not part of any known subfamily of the *Autographiviridae* family (Figure 6). To confirm our assumption, the level of intergenomic similarity between the phages of this clade was also calculated and compared with a similar indicator in other subfamilies. It was found that the studied group of phages had an SG level of >13% (Figure 7). This corresponded to the SG calculated for several other subfamilies of the *Autographiviridae* family (Figure 7). 

To confirm our suggestion, the phylogenetic analysis of two essential proteins of StenM_174, namely the capsid protein and the large subunit of terminase, was carried out (Supplementary, Figures S2 and S3). It was found that the topology of the protein phylogenetic trees corresponded to the results of comparative genomic analysis.

Consequently, the studied group of phages can be combined into the proposed subfamily *Pradovirinae*.

## 4. Discussion

The isolation and characterization of phages specific to the multidrug-resistant pathogen *S. maltophilia* open new therapeutic possibilities. The search for new phages with therapeutic potential is important because of the high genetic and phenotypic heterogeneity of the *S. maltophilia* populations [3]. In this study, we isolated a new phage, which infects *Stenotrophomonas* spp. isolates, and we characterized this phage in terms of genome, taxonomy, and biological properties. The studied phage possessed high lytic activity, and its virions corresponded to the morphotype of podoviruses. A comparative analysis of the genome revealed that the *Stenotrophomonas* phage StenM_174, along with the phages Ponderosa, Pepon, Ptah, and TS-10, can be members of the new putative genus *Ponderosavirus* within the *Autographiviridae* family. In addition, the data analysis suggests a new putative subfamily, *Pradovirinae*, within the *Autographiviridae* family. 

Members of the *Autographiviridae* family have outstanding antibacterial potential since most of them are strictly lytic and highly virulent for their hosts. This type of phage often uses surface lipopolysaccharides of bacterial cells as receptors. Due to a wide variety of surface polysaccharides in Gram-negative bacteria, most podoviruses are highly specific and have a narrow host range, which is a bottleneck for the use of this group of phages as antibacterial agents. Note that multistep bacterial killing experiments revealed the lytic characteristics of the StenM_174 phage and its potential for use in the treatment of infections caused by Stenotrophomonas (Figure 2). At the same time, the regrowth of phage-resistant bacteria was observed several hours after exposure to the phage, so using a specific phage may not be sufficient. Therefore, the preferred method of phage therapy is to apply a phage cocktail, which reduces the growth of phage-resistant cells, or to combine phage treatment with antibiotics. 

In fact, there is limited information about the host range for phages that are similar to StenM_174. Currently, the bacterial host range has been reported for only two phages, BUCT598 and BUCT609. The first reproduced in 30%, and the second reproduced in 38% of the tested strains [19,50]. Surprisingly, the studied phage StenM_174 reproduced well in a wide range of *S. maltophilia* strains (>70% of the tested strains) and several strains of other *Stenotrophomonas* species. This phage likely uses a conservative part of the surface lipopolysaccharide as a receptor. In addition, the genome of the phage contains two genes encoding receptor-binding proteins, namely a tail fiber adapter (gp43) and tail fiber (gp44), each of which can interact with different bacterial receptors. This contributes to the expanded number of susceptible *Stenotrophomonas* strains.

Despite the large number of recently isolated and characterized *Stenotrophomonas* phages, little is known about their phage–host interaction and their attachment to bacterial receptors. So far, only two types of bacterial receptors have been identified. The first is the TonB protein, which interacts with the *Stenotrophomonas* phage BUCT603 [20]. The second receptor is Type IV bacterial pili. The latter are essential receptors for a group of similar *Stenotrophomonas* phages [51,52,53]. In this regard, further identification of the receptor-binding proteins of these phages and the corresponding bacterial surface receptors is of interest.

## Figures and Tables

**Table 1:** List of studied *Stenotrophomonas* strains.

No	Species ^1^	Number of Environmental Strains	Number of Strains Isolated from Insects	Number of ClinicalStrains
1	*S. maltophilia*	4 (3) ^2^	4 (4)	14 (9)
2	*S. rhizophila*	15 (1)	0	0
3	*S. geniculata*	2	0	4
4	*S. pavanii*	5	0	1
5	*S. chelatiphaga*	5	0	0
6	*S. lactitubi*	5 (1)	0	0
7	*S. bentonitica*	2	0	0
8	*S. acidaminiphila*	1	0	0
9	*S. tumulicola*	1	0	0
10	*S. nematodicola*	1	0	0
	Total	41 (5)	4 (4)	20 (9)

^1^ Bacterial strains were identified using 16S rRNA gene sequencing. ^2^ The number of strains sensitive to the phage StenM_174 is indicated in brackets.

**Table 2:** List of *Stenotrophomonas* strains sensitive to StenM_174.

No	Species	Isolation Source	No in CEMTC (GenBank ID)	Growth Temperature, °C	Relative Efficiency of Plating (EOP) ^2^
1	*S. lactitubi*	environmental	1947 (MZ424758)	25	high
2	*S. maltophilia*	environmental	2142 (MZ424754)	25	high
3	*S. maltophilia*	environmental	3963 (MZ424759)	25	high
4	*S. maltophilia*	environmental	7824	25	high
5	*S. maltophilia*	clinical	2164 (MZ424760)	37	low
6	*S. maltophilia*	clinical	2329 (MZ424765)	37	low
**7**	***S. maltophilia* ^1^**	**clinical**	**2355 (OP393915)**	**37**	**5 × 10^7^**
8	*S. maltophilia*	clinical	2356	37	high
9	*S. maltophilia*	clinical	2517 (MZ424766)	37	low
10	*S. maltophilia*	clinical	3051 (MZ424761)	37	low
11	*S. maltophilia*	clinical	3806 (MZ424764)	37	low
12	*S. maltophilia*	clinical	4225	37	medium
13	*S. maltophilia*	clinical	4227	37	high
14	*S. maltophilia*	insect	3659 (MT040043)	28	medium
15	*S. maltophilia*	insect	3664 (MT040044)	28	high
16	*S. maltophilia*	insect	3670 (MT040045)	28	medium
17	*S. maltophilia*	insect	3672 (MT040046)	28	medium
18	*S. rhizophila*	environmental	5507 (OQ353079)	25	low

^1^ Bacterial host/isolation strain marked with bold. ^2^ The EOP value = phage titer on test bacterium/phage titer on host bacterium. EOP values of >1 were ranked as ‘high’ efficiency; 0.2–1 denoted ‘medium’ efficiency; and 0.001–0.2 denoted ‘low’ efficiency.

## Data Availability

The genome sequence of the *Stenotrophomonas* phage StenM_174 and 16S rRNA sequences of *Stenotrophomonas* strains are available in the GenBank database.

## Supplementary Materials

The following supporting information can be downloaded at: https://www.mdpi.com/article/10.3390/v16010018/s1, Data S1. Data on Stenotrophomonas phages extracted from the GenBank database. Data S2. Analysis of the StenM_174 genome using PhageTerm tool. Data S3. Analysis of the protein sequence of the StenM_174 endolysin using the SignalP tool. Figure S1. AlphaFold models are colored according to the modeling confidence. Figure S2. Phylogenetic analysis of the major capsid protein was carried out using MEGA 7.0. Sequence alignment was performed using CLUSTALW; the maximum likelihood method with the bootstrap value 1000 was used for phylogenetic tree construction. The putative *Pradovirinae* subfamily is marked with a light-red box, and *Okabevirinae* and *Melnykvirinae* are marked with blue and yellow boxes, respectively. Figure S3. Phylogenetic analysis of the large subunit of terminase was carried out using MEGA 7.0. Sequence alignment was performed using CLUSTALW; the maximum likelihood method with the bootstrap value 1000 was used for phylogenetic tree construction. The putative *Pradovirinae* subfamily is marked with a light-red box, and *Okabevirinae* and *Melnykvirinae* are marked with blue and yellow boxes, respectively.
