# Supplementary figures and images for: StenM_174: A Novel Podophage That Infects a Wide Range of Stenotrophomonas spp. and Suggests a New Subfamily in the Family Autographiviridae
Source: Viruses. 2023 Dec 21;16(1):18. doi: 10.3390/v16010018 (PMC10820202; doi:10.3390/v16010018)

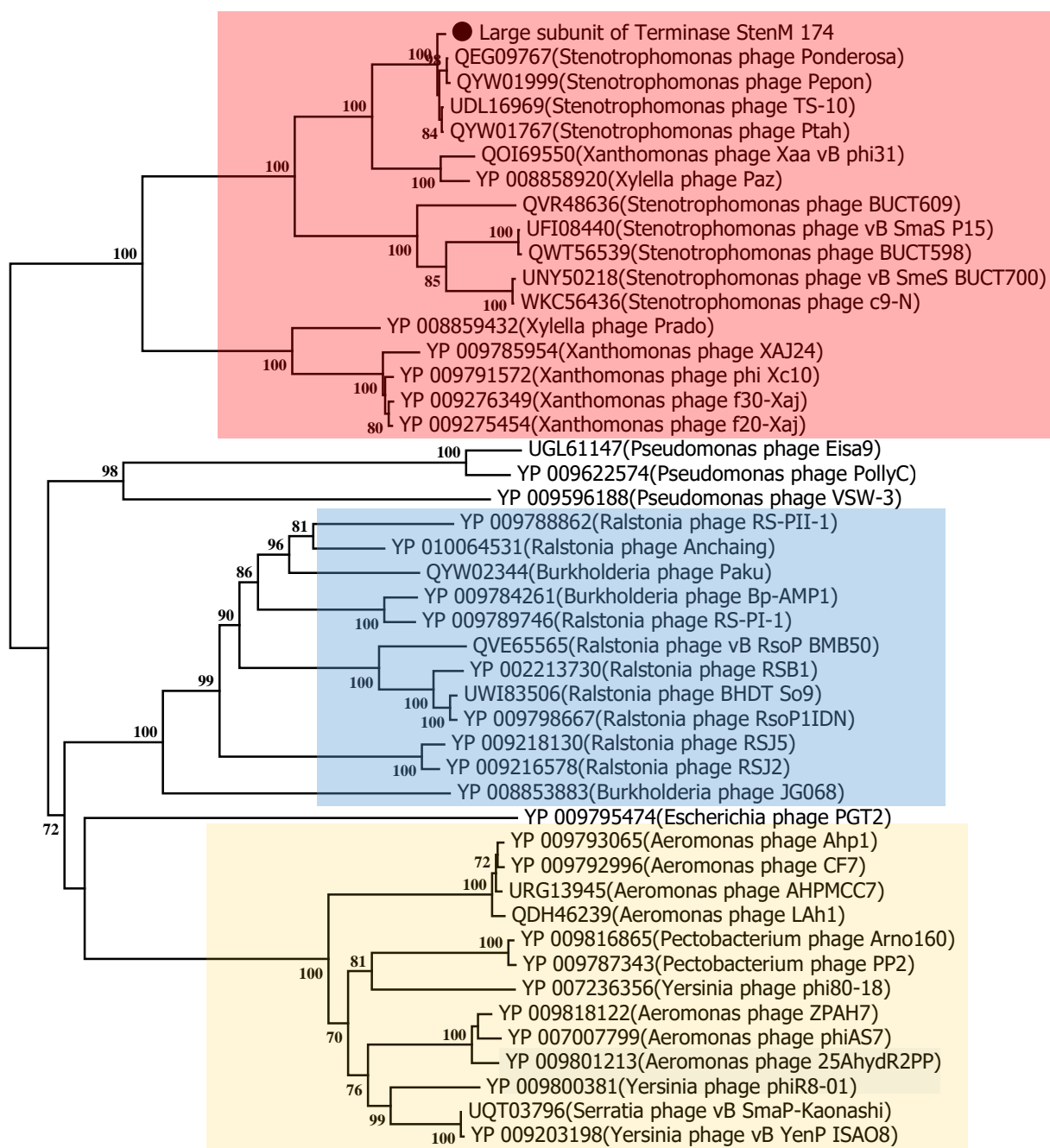

0.1

Supplement: Supplementary file 1 [file viruses-16-00018-s001.zip › Figure S3.pdf]

SignalP 6.0 prediction: Sequence

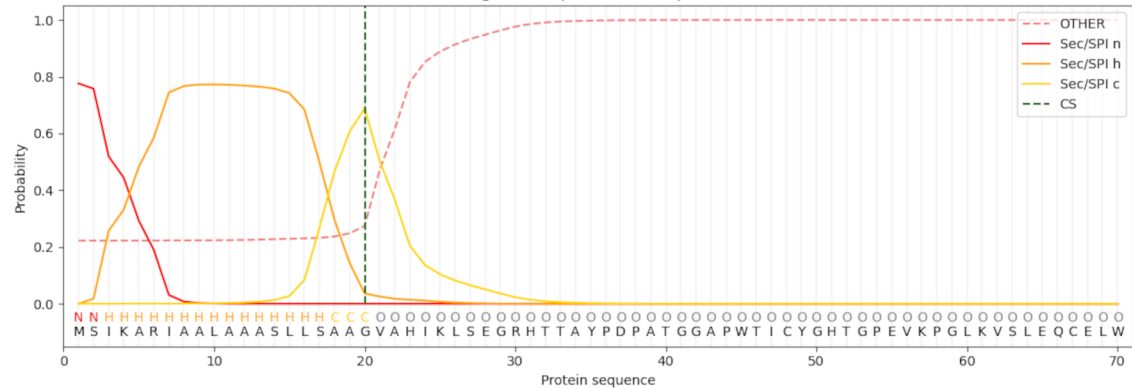

Supplement: Supplementary file 1 [file viruses-16-00018-s001.zip › Data S3.pdf]

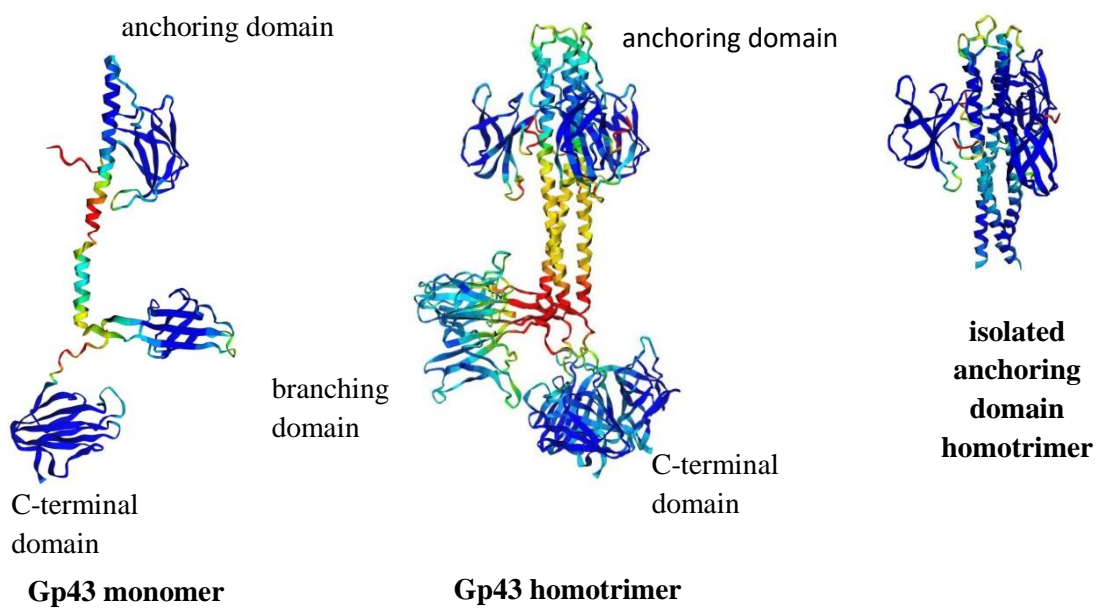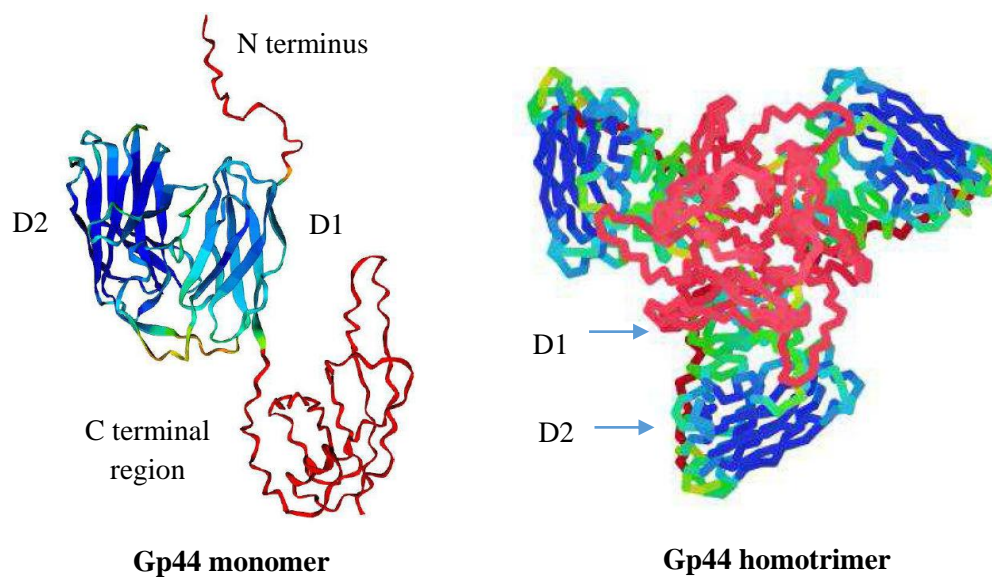

pI/DDT: ■ Very low (<50) ■ Low (60) ■ OK (70) ■ Confident (80) ■ Very high (>90)

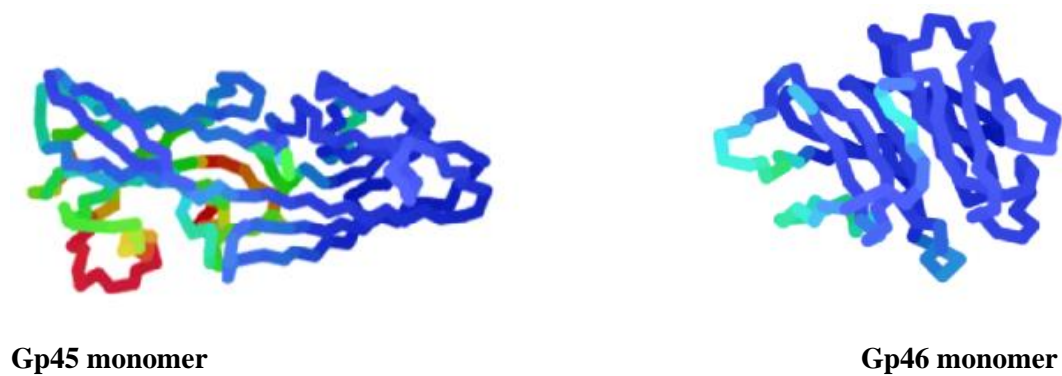

Supplement: Supplementary file 1 [file viruses-16-00018-s001.zip › Figure S1.pdf]

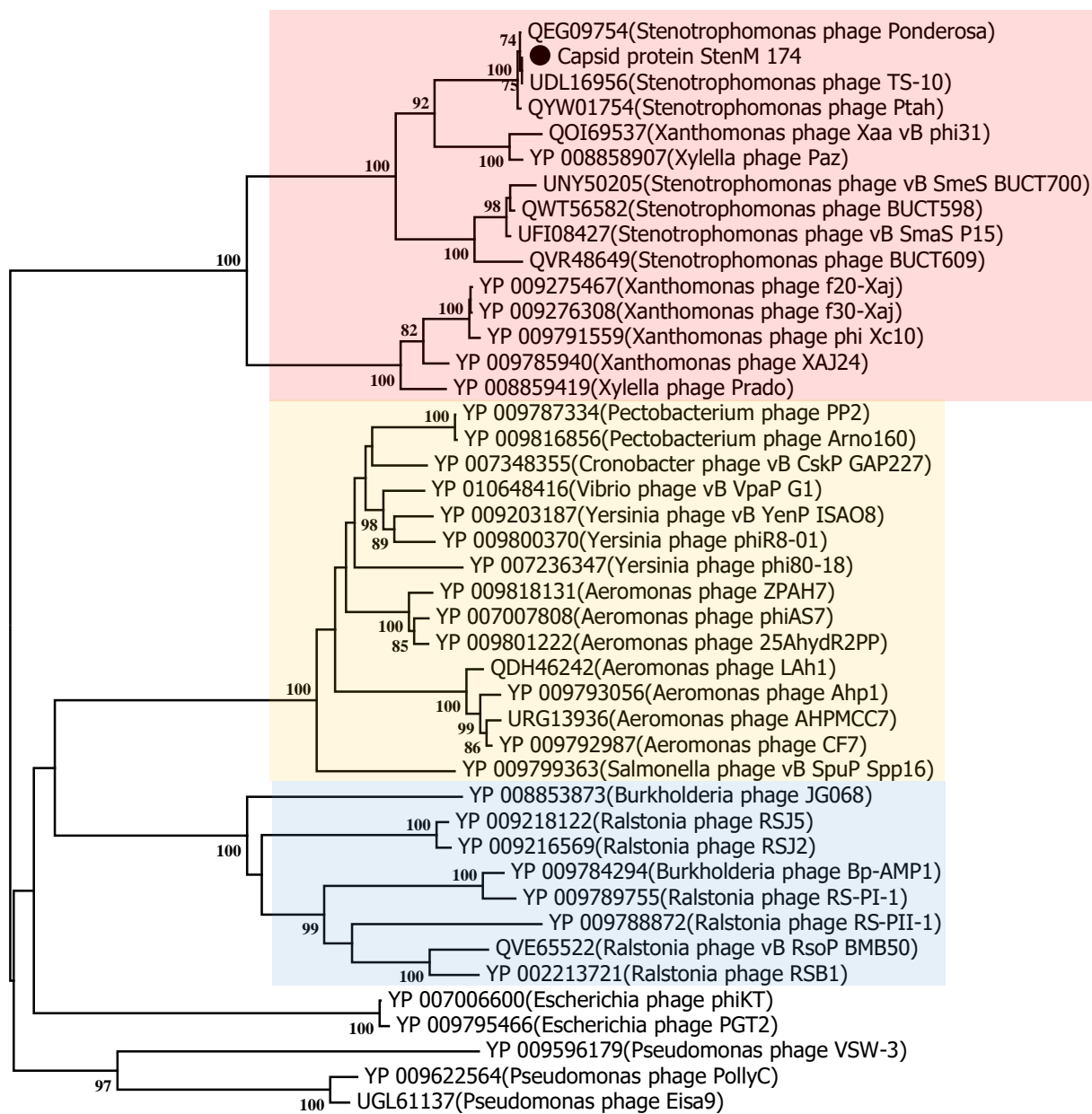

0.1

Supplement: Supplementary file 1 [file viruses-16-00018-s001.zip › Figure S2.pdf]
